# Study of Anti-Fatigue Effect in Rats of Ferrous Chelates Including Hairtail Protein Hydrolysates

**DOI:** 10.3390/nu7125504

**Published:** 2015-12-01

**Authors:** Saibo Huang, Huimin Lin, Shang-gui Deng

**Affiliations:** 1 School of Marine Science and Technology, Zhejiang Ocean University, No.1, Haida South Road, Lincheng Changzhi island, Zhoushan 316022, China; 15257075373@163.com; 2 School of Food and Medicine, Zhejiang Ocean University, No.1, Haida South Road, Lincheng ChangzhiIsland, Zhoushan 316022, China; linhuixiaomin@126.com

**Keywords:** hairtail protein peptide, ferrous ion, chelate, anti-fatigue, rat

## Abstract

The ability of ferrous chelates including hairtail protein hydrolysates to prevent and reduce fatigue was studied in rats. After hydrolysis of hairtail surimi with papain, the hairtail protein hydrolysates (HPH) were separated into three groups by range of relative molecular weight using ultrafiltration membrane separation. Hairtail proteins were then chelated with ferrous ions, and the antioxidant activity, the amino acid composition and chelation rate of the three kinds of ferrous chelates including hairtail protein hydrolysates (Fe-HPH) were determined. Among the three groups, the Fe-HPH chelate showing the best conditions was selected for the anti-fatigue animal experiment. For it, experimental rats were randomly divided into seven groups. Group A was designated as the negative control group given distilled water. Group B, the positive control group, was given glutathione. Groups C, D and E were designated as the Fe-HPH chelate treatment groups and given low, medium, and high doses, respectively. Group F was designated as HPH hydrolysate treatment group, and Group G was designated as FeCl_2_ treatment group. The different diets were orally administered to rats for 20 days. After that time, rats were subjected to forced swimming training after 1 h of gavage. Rats given Fe-FPH chelate had higher haemoglobin regeneration efficiency (HRE), longer exhaustive swimming time and higher SOD activity. Additionally, Fe-FPH chelate was found to significantly decrease the malondialdehyde content, visibly enhance the GSH-Px activity in liver and reduce blood lactic acid of rats. Fe-HPH chelate revealed an anti-fatigue effect, similar to or better than the positive control substance and superior to HPH or Fe when provided alone.

## 1. Introduction

Fatigue is a complex physiological and biochemical process in the human body. It is also a very common social problem at present since the fierce competition in modern society places people under enormous pressure. Further, modern lifestyle is becoming more and more irregular, this leading people more vulnerable to fatigue. Accordingly, many efforts have been addressed towards the development of anti-fatigue products [1].

Hairtail (*Trichiurus lepturus*), an aquatic product, is rich in protein content and is considered as highly nutritious. It is mainly distributed in the Chinese Yellow Sea, East China Sea, and Bohai Sea, showing a high annual output. It is considered one of the Four Chinese Seafoods, alongside with the large yellow croakers (*Larimichthy scrocea*), small yellow croakers (*Larimichthys polyactis*) and cuttlefish (Sepiidae); however, its cost is far lower than that of such other products, so that it can be considered more accessible to most consumers.

With the constant refinement and optimization of enzymatic hydrolysis conditions, research into enzymatic hydrolysis protein peptides has become more and more abundant, and the employment of active peptides has attracted a great attention. Thus, different kinds of polypeptides are known to have anti-oxidant, anti-bacterial, anti-tumor, and anti-hypertensive activities [2,3,4,5,6,7]. Among such profitable effects, anti-fatigue activity should also be considered. In this sense, Fang *et al.* [1] employed three types of hydrolytic enzymes to hydrolyze long oyster meat to obtain its hydrolysates; as a result, all kinds of hydrolysates provided good anti-fatigue effects. Shi *et al.* [8] obtained a hydrolysate by enzymatically hydrolyzing danio Spanish mackerel; through further experiments concerning mice feeding, this hydrolysate showed to have significant anti-fatigue effects. Kai Xu *et al.* [9] focused on the effect of defatted protein peptides from Antarctic krill; after intragastric administration in mice, they found it could significantly improve the anti-fatigue ability.

Iron is the most prevalent trace element in the human body; its deficiency in human being can lead to anemia, so that iron supplements are widely used. Previous studies have shown that iron deficiency can also decrease productivity at work and exercise capacity and endurance in sports [10,11], this indicating that iron plays a role in promoting the resistance to fatigue. Based on the above two points, it was guessed that if fish protein peptides were chelated with ferrous ions, the ferrous-chelating fish peptides may have better anti-fatigue effect. Chelating agents, such as peptides, have been reported to decrease the free iron content and increase the iron bioavailability [12]. Protein hydrolysate fractions with ferrous chelating enhanced iron solubility capabilities and iron bioaccessibility [13].

In the present study, therefore, the effect of hairtail protein peptides chelated with Fe^2+^ was encountered; in it, different hairtail peptide fractions were obtained and subsequently chelated with ferrous ions, being the anti-fatigue effects of the chelates analyzed. A theoretical basis for further development and use of ferrous peptide chelates is discussed and proposed.

## 2. Materials and Methods

### 2.1. Materials and Processing

Hairtail surimi was provided by the Zhejiang Xingye Co., Ltd. (Zhoushan, China). Frozen surimi portions (100–200 g) were packed into plastic bags and placed in a −20 °C freezer until required for processing; at that time, surimi samples were thawed overnight in a refrigerator (4 °C).

### 2.2. Reagents

Papain (20,000 IU/g enzyme activity) was purchased from Shanghai Jinsui Biological Science and Technology Co., Ltd. (Shanghai, China); deoxyribose, thiobarbituric acid and glutathione were purchased from Sigma Co., Ltd. (Shanghai, China); ferrous chloride (food grade), ascorbic acid, sodium hydroxide, and other analytical reagents were purchased from Sinopharm Group Pharmaceutical Co., Ltd. (Beijing, China); detection kits for superoxide dismutase (SOD), malondialdehyde (MDA), glutathione peroxidase (GSH-PX), and lactic acid were purchased from Nanjing Jiancheng Biology Engineering Research (Nanjing, China).

### 2.3. Main Instruments and Equipment

The following were employed: 1812G membrane separation equipment (Hangzhou Watech Membrane Engineering Co., Ltd., Hangzhou, China), FD-1000 freeze dryer (Tokyo, Japan), Spectra Plus 384 multiskan spectrum (California, MD, USA), L-8900 automatic amino acid analyzer (Hitachi Co., Ltd, Tokyo, Japan), Sysmex K-1000D automated haematology analyzer (Kobe, Japan), U-2800 ultraviolet spectrophotometry (Hitachi Co., Ltd., Tokyo, Japan).

### 2.4. Preparation of Ferrous Chelates with Hairtail Protein Hydrolysates (Fe-HPH)

#### 2.4.1. Preparation of the Hairtail Protein Hydrolysate (HPH)

The hydrolysate was prepared in the laboratory. Optimized conditions of enzymatic hydrolysis were as follows: 1:10 (material to liquid ratio), 6.5 (pH), 45 °C (enzymolysis temperature), and 8 h (enzymolysis time). During enzymatic hydrolysis, the pH of solution was kept at about 6.5 by adding 1 mol/L HCl/NaOH solution every 2 h. For the enzyme inactivation, a 90 °C-heating for 20 min was applied.

#### 2.4.2. Separation and Fractionation of the Hydrolysate (HPH)

The hydrolysate was filtered with filter paper and with 0.45 μm and 0.22 μm microporous membranes to produce a clear and transparent liquid. Ultrafiltration membranes of different intercept molecular weights (1 ku and 5 ku) were used to fractionate the hydrolysate and produce three hydrolysates with different peptide fragments. Such hydrolysates were named as hairtail protein hydrolysate I (HPH I; relative molecular mass < 1 ku), hairtail protein hydrolysate II (HPH II; relative molecular mass 1–5 ku), hairtail protein hydrolysate III (HPH III; relative molecular mass >5 ku). After vacuum concentration and freeze drying, powders corresponding to all three hydrolysates were obtained susceptible to be employed in the following steps.

#### 2.4.3. Chelation of HPH and Ferrous Ions

The three above mentioned kinds of hairtail protein peptides were chelated with Fe^2+^ respectively. For it, FeCl_2_ (1 M solution) was used as the ferrous source. The pH of HPH solution was kept at 7.0 by adding 0.1% ascorbic acid and the mixture placed in a water bath with constant temperature (30 °C) for 15 min. Then, FeCl_2_ solution was added at a ratio of 1 (FeCl_2_ solution): 50 (enzymolysis liquid) (*v*/*v*) ratio, being the mixture stirred for 30 min. After that time, the reaction media were vacuum concentrated and freeze-dried to produce three different powders of Fe-HPH chelates (Fe-HPH I, Fe-HPH II, and Fe-HPH III, respectively).

### 2.5. Determination of Fe-HPH Chelates

#### 2.5.1. Chelating Rate

Free ferrous ion content was determined using the 1,10-phenanthroline method [14].

Total ferrous iron chelating rate was calculated as the following ratio: (total ion content—free ferrous ion content)/total number of ferrous ions.

#### 2.5.2. Amino Acid Composition

The amino acid composition of the three kinds of ferrous chelate powders of hairtail protein peptides (Fe-HPH I, Fe-HPH II, and Fe-HPH III) was determined using an automatic amino acid analyzer [15].

#### 2.5.3. *In Vitro* Antioxidant Activity

Different methods were applied such as hydroxyl, DPPH (2,2-Diphenyl-1-picrylhydrazyl) and superoxide anion radical scavenging activities, according to the method of Lin *et al.* [16], with some modifications. For it, the same mass-volume concentration (4 mg/mL) of Fe-HPH I, Fe-HPH II and Fe-HPH III chelates was prepared, being the same glutathione (GSH) solution (4 mg/mL) used as control. All assays were carried out in triplicate.

#### 2.5.4. Fe Content

The Fe content of Fe-HPH chelates was determined using atomic absorption spectroscopy [17]. All assays were carried out in triplicate.

### 2.6. Anti-Fatigue Animal Experiments

Seventy weaned Wistar rats (50 ± 2 g, specific pathogen-free grade, SPF), half male and half female, were provided by Shanghai Slake Laboratory Animal Co., Ltd. (Shanghai, China). The experimental procedures and the animal use and care protocols were approved by the Committee on Ethical Use of Animals of Zhejiang Ocean University (Project code: 2014AE043; Date of approval: 11 March 2014).

The rats were randomly divided into seven groups. Group A was designated as the negative control group given the same volume distilled water by gavage every day; Group B was designated as the positive control group given glutathione (100 mg/kg·bw) by gavage every day; Groups C, D, and E were designated as the Fe-HPH chelates treatment groups and given low, medium, and high doses (50, 100, 200 mg/kg·bw) by gavage every day, respectively; Group F was designated as the HPH hydrolyzate treatment group (200 mg/kg·bw) by gavage every day, maintaining the same peptide content of Group E; Group G was designated asthe FeCl_2_ treatment group, being the Fe content the same than that of Group E. Each group was half male and half female. Dry powder was first dissolved in distilled water, concentrated, and given by gavage. All groups were daily fed by gavage at the same time of the day, and the rats were subjected to 30 min swimming training for adaption after 1 h of gavage. This treatment was continued for 20 day. Body weight was recorded at the beginning (on day 0) and at the end (after gavage for 12 h on day 20) of the experiment. Blood was collected from the caudal vein to determine hemoglobin (Hb) content.

During the experiment, the rats were housed under the following conditions: 22 ± 2 °C temperature, 50% ± 5% relative humidity, a 12 h light and 12 h dark cycle, and less than 60 dB noise. All seven groups of rats were allowed free access to normal food (Fe content: 35 mg·kg^−1^, provided by the Animal Experimental Research Center of Zhejiang province) and distilled water. Food intake of each rat was measured every day.

#### 2.6.1. Calculation of Haemoglobin Regeneration Efficiency (HRE)

HRE value was calculated according to the method of Zhang [18]:

(1) $$Hb\cdot Fe\;(mg)=body\text{​ }weight\;(g)\times \text{​ }0.067\times Hb\;(g\cdot mL^{-1})\times \;3.35$$

where 0.067 is referred to the volume (in milliliters) of blood per gram of body weight; 3.35 is referred to the Fe content (in milligrams) per gram of Hb. HRE was also calculated as percentage according to the following formula:

(2) $$HRE\;\%=\frac{Hb\cdot Fe\;(final)-Hb\cdot Fe\;(initial)}{Fe\;intake}\times \;100$$

where Fe intake (in milligrams) was the total Fe content including normal food and intragastric materials.

#### 2.6.2. Determination of Exhaustive Swimming Time of Rats

The exhaustive swimming exercise was conducted after 1 h of gavage on day 20. The rats were placed in a 25 ± 1 °C water tank that was 35 cm water deep (90 cm × 45 cm × 45 cm). The water was stirred with a glass rod to make the rats keep moving. Swimming time was recorded, *i.e.*, time from the mouse entering water to the rats’ heads sinking completely below the surface of the water for 6 s and not rising again.

#### 2.6.3. Detection of SOD, MDA, GSH-Px and Blood Lactic Acid

Exhausted rats were allowed to rest for 30 min. They were then sacrificed in decapitation. Their blood was collected, and heparin was added to prevent coagulation. Homogenates were prepared from the hindlimb muscle and livers of rats using an ice saline bath. SOD activity and MDA content were detected in whole blood, liver tissue, and muscle tissue and GSH-Px activity was detected in liver tissue and blood lactate indices according to the kit instructions.

#### 2.6.4. Statistical Analysis

All tests were conducted in triplicate. Experimental results are showed as mean ± standard deviation and subjected to one-way analysis of variance. Values of *p* < 0.05 were regarded as significant.

## 3. Results and Discussion

### 3.1. Analysis of the Amino Acid Composition and Chelating Rate

The bioactivities of protein hydrolysates often depend on amino acid composition, concentration, and sequence. Three kinds of Fe-HPH amino acid compositions are showed in Table 1. There have been previous studies pointing out that threonine, serine, glycine, alanine, valine, leucine, and tyrosine [19] contents could decrease rapidly in human endurance tests. This decrease may be related to fatigue, so if the seven amino acids are present in the diet at a high concentration, exercise capacity might be increased and fatigue might be reduced. Additionally, lysine, histidine, methionine, cysteine and phenylalanine have showed antioxidant activity or to be closely concerned with promoting the generation of antioxidant activity [20]. Accordingly, the presence of high concentrations of such amino acids would lead to free radicals removing and consequently, prevention of their generation would reduce fatigue. Further, glutamic acid was found to have a positive effect on the nervous system [21], and aspartic acid showed to reduce the concentration of ammonia in the blood concentration [22]; as a result, the employment of both amino acids would be able to relieve fatigue. In the present research, contents on the seven amino acids related to anti-fatigue activity accounted for 46.32%, 39.43%, and 38.22% of all amino acids in the Fe-HPH I, Fe-HPH II, and Fe-HPH III groups, respectively. The five amino acids relating to antioxidant activity accounted for 14.41%, 19.11%, and 19.75%, respectively. The proportions of glutamic acid and aspartic acid were 14.95%, 26.23%, and 25.30%, respectively, and 8.65%, 6.71%, and 4.64%, respectively. The proportion of anti-fatigue amino acids was highest in the Fe-HPH I chelate, but its proportion of antioxidant amino acids was lowest when compared with its counterpart hydrolysates. Meantime, Fe-HPH III chelate showed the lowest proportion of anti-fatigue amino acids and the highest proportion of antioxidant amino acids. While the amino acid concentrations related to anti-fatigue and anti-oxidation in Fe-HPH II chelate were both moderate, those of glutamic acid and aspartic acid were both high, suggesting that Fe-HPH II chelate might have a best potential anti-fatigue effect.

**Table 1:** Amino acid composition of three kinds of Fe-hairtail protein hydrolysates (HPH) chelate (μg/mL).

Amino Acid	Fe-HPH I	Fe-HPH II	Fe-HPH III
Aspartic acid	6.147	6.032	2.788
Threonine	3.007	1.769	0.983
Serine	2.783	1.628	0.946
Glutamic acid	10.622	23.585	15.184
Glycine	12.710	11.702	6.858
Alanine	7.246	9.741	6.390
Cysteine	0.567	3.900	2.898
Valine	2.989	4.979	3.423
Methionine	1.547	1.864	1.139
Isoleucine	2.121	3.101	2.109
Leucine	4.181	5.637	4.345
Tyrosine	0.000	0.000	0.000
Phenylalanine	2.389	3.901	2.686
Lysine	4.423	7.274	5.131
Histidine	1.312	0.239	0.000
Arginine	5.372	1.638	0.911
Proline	3.648	2.920	4.234

### 3.2. In Vitro Antioxidant Activities of Fe-HPH Chelates

The antioxidant activity of Fe-HPH chelates was evaluated using free radical scavenging activity assays. Hydroxyl and superoxide anion radicals can easily react with and/or oxidize biomolecules, such as carbohydrates, proteins and DNA, leading to oxidative stress, cell injury and physiological disorders, all these damages leading to functions destruction in human body. On the other side, DPPH method has extensively been used to evaluate the formation of reducing substances [22]. As shown in Table 2, Fe-HPH II chelate had strong scavenging activities on hydroxyl, DPPH and superoxide anion radicals, being significantly higher (*p* < 0.05 or *p* < 0.01) than those of the other two kinds of Fe-HPH chelates. Furthermore, Fe-HPH II exhibited stronger *in vitro* scavenging activities towards both DPPH (*p* < 0.01) and superoxide anion radicals (*p* < 0.05) than glutathione (GSH). Free radical scavengers such as phenolic and carotenoid compounds have shown to prevent oxidative stress and further enhance recovery from fatigue [21]. In the present study, Fe-HPH II chelate showed to possess a very high radical scavenging activity, this suggesting a beneficial role in order to alleviate physical fatigue.

**Table 2:** *In vitro* antioxidant activity of three kinds of Fe-HPH chelates and glutathione (GSH).

Sample	Antioxidant Activity (%)		
	Hydroxyl Radical Scavenging	DPPH Radical Scavenging	Superoxide Anion Radical Scavenging
Fe-HPH I	27.23 ± 0.35 **	23.71 ± 0.96 **	18.21 ± 0.77 **
Fe-HPH II	63.14 ± 1.55	75.88 ± 2.04	47.35 ± 0.96
Fe-HPH III	46.63 ± 1.72 *	55.93 ± 2.25 *	33.12 ± 1.88 *
GSH	60.06 ± 2.17	12.37 ± 1.33 **	40.05 ± 1.93 *

Note: Relative to Fe-HPH II chelate, * *p* < 0.05; ** *p* < 0.01.

Moreover, present results also confirmed the above-mentioned amino acid composition. Thus, Table 3 provides the comparative chelating rates of the three kinds of chelates. As a result, Fe-HPH II chelate showed to include the greatest number of ferrous ions (relative molecular mass 1–5 ku; Fe content 6.01 g/kg). Consequently, this kind of chelate should be selected for the subsequent animal experiments to be carried out in the present research. For it, and according to the experimental part description, three different groups corresponding to low, medium and high doses of Fe-HPH II chelate (Groups C, D and E) were encountered; meantime, HPH II hydrolysate was designated as Group F.

**Table 3:** The chelating rate of three kinds of Fe-HPH chelates.

Kind	Fe-HPH I	Fe-HPH II	Fe-HPH III
Chelating rate (%)	64.32	75.96	70.88

### 3.3. Fe-HPH II Chelate and Haemoglobin Regeneration Efficiency (HRE)

HRE is regarded as a testing method for iron bioavailability assessment [23,24]; its value is positively correlated with the iron bioavailability, while iron presence would develop an important role related to fatigue resistance as described previously [10,11]. Consequently, HRE value would indirectly reflect the anti-fatigue abilities of different samples, so that the higher the HRE value, the stronger the ability of fatigue resistance. The calculation of the HRE value involved three data including body weight, haemoglobin content and Fe intake of rats described previously. As shown in Table 4, food intake of rats did not provide significant differences as a result of the diet provided. As shown in Table 5, on day 0, the body weight and haemoglobin content of rats in each group had no significant difference. On day 20, the body weight of rats in Group E was significantly higher (*p* < 0.05) than that in Group A (negative control group). This can be explained as a result of the combination effect of the ferrous ion and the active peptide, being both of them necessary for healthy body growth. The value of Group F was relatively low but very close to that of Group E, which indicated that active peptides played a greater role in body growth. The haemoglobin contents of rats in Groups D, E and G were significantly higher (*p < 0.05* or *p < 0.01*) than that in Group A.

**Table 4:** Fe intake of rats.

Group	Food Intake/g/rat·Day	Fe Intake in Food/mg/rat·Day	Fe Intake by Gavage/mg/kg·bw
A	13.52 ± 1.20	0.47 ± 0.04	0
B	15.31 ± 2.56	0.54 ± 0.09	0
C	14.25 ± 1.93	0.50 ± 0.07	3.01
D	15.36 ± 2.40	0.54 ± 0.08	6.01
E	16.20 ± 3.88	0.57 ± 0.07	12.02
F	14.40 ± 0.56	0.51 ± 0.02	0
G	14.72 ± 0.37	0.52 ± 0.01	12.02

HRE value was affected by the diet and by anemia [17]. Rats in this study were all Fe-normal, food intake of every group was almost equal; consequently, HRE score was only affected by the materials of intragastric administration. As shown in Table 5, HRE value of Group E (given high dose of Fe-HPH chelate) was high, showing to be significantly higher (*p* < 0.01) than that of Group A. Group D had a significant difference (*p* < 0.05) when compared with individuals corresponding to Group A. Mean HRE values of Groups C and G were slightly higher than those of Group A, while individuals belonging to Group F (HPH hydrolysate diet) showed the lowest scores. Furthermore, there was a significant difference (*p* < 0.05) in HRE value between Group E and Group B (positive control group). It was concluded that the increasing effect on the HRE value of individuals corresponding to Fe-HPH chelate intake was superior to that of Fe or HPH hydrolysate alone, indirectly indicating that Fe-HPH chelate had a stronger anti-fatigue effect.

**Table 5:** Body weight, hemoglobin (Hb) content and haemoglobin regeneration efficiency (HRE) of rats at the beginning and end of experiments.

Group	Body Weight g		Hb Content g·L		HRE %
	0 Day	20 Day	0 Day	20 Day	
A	160.32 ± 4.11	235.33 ± 7.16	135.24 ± 2.12	134.33 ± 1.92	58.74 ± 4.20
B	158.87 ± 5.83	238.77 ± 6.25	134.76 ± 1.55	136.61 ± 2.02	59.51 ± 3.89
C	163.19 ± 5.46	237.78 ± 4.33	135.33 ± 3.52	139.87 ± 4.26	60.45 ± 6.22
D	160.28 ± 4.07	239.15 ± 5.28	138.12 ± 2.08	144.66 ± 3.13 *	63.15 ± 6.01 *
E	157.65 ± 6.09	245.41 ± 5.16 *	136.43 ± 3.36	149.72 ± 5.21 *^,∆^	76.23 ± 7.67 **^,∆^
F	161.79 ± 5.12	243.88 ± 3.39	133.50 ± 4.28	134.69 ± 2.45	56.30 ± 4.30
G	160.34 ± 5.30	239.52 ± 6.94	135.11 ± 3.01	142.77 ± 3.32 *	59.22 ± 3.88

Note: Relative to group A, * *p* < 0.05; ** *p* < 0.01; compared with group B, ^∆^ *p* < 0.05.

### 3.4. Fe-HPH II Chelate and Exhaustive Swimming Time

The improvement in exercise endurance is the most direct and objective indicator of enhanced anti-fatigue ability. The length of exhaustive swimming time can indicate the degree of fatigue. As shown in Table 6, the rats in all groups had longer exhaustive swimming time than that in Group A (negative control group). Among these groups, Group E prolonged the exhaustive swimming time extremely significantly (*p* < 0.01), Groups D and G prolonged significantly (*p* < 0.05), while Groups C and F had a tendency of prolonging, but not significant. The exhaustive swimming time of rats in Groups C, D and E, which were given Fe-HPH chelates, gradually extended with the increase of dose; the exhaustive swimming time of individuals belonging to Group E was visibly higher (*p* < 0.05) than that of their counterparts from Group B (positive control group). As a conclusion, exhaustive swimming time of rats given Fe-HPH chelate was longer than that of rats given HPH or Fe alone.

**Table 6:** Exhaustive swimming time of rats.

Group	Number of Animals	Exhaustive Swimming Time (Min)
A	10	98.20 ± 18.62
B	10	134.25 ± 18.93 *
C	10	109.27 ± 10.57
D	10	130.11 ± 11.80 *
E	10	166.34 ± 23.42 **^,∆^
F	10	103.27 ± 18.04
G	10	119.15 ± 9.43 *

Note: Relative to group A, * *p* < 0.05; ** *p* < 0.01; compared with group B, ^∆^ *p* < 0.05.

### 3.5. Fe-HPH II Chelate and SOD Activity in Rats

SOD is an important antioxidant enzyme; it prevents damage produced by free radicals. When the body has a large amount of SOD, it can undergo disproportioned catalysis of free radicals to scavenge free radicals, reduce free radical damage to cells, delay fatigue, and accelerate recovery after a fatigue stage [25].

As shown in Table 7, in whole blood, the SOD activities of rats in Group B (positive control group) and Groups D and E were significantly or extremely significantly (*p* < 0.05 or *p* < 0.01) higher than those in Group A (negative control group). The SOD activities in individuals from Groups C, F and G did not increase significantly, although the following decreasing sequence could be depicted: Group C > Group F > Group G. Except for Group B individuals, a significant effect (*p* < 0.05) in the liver could be obtained. The SOD activities in other test groups (Groups C, D, E, F and G) did not significantly differ from those in Group A. The SOD activities in Groups C, D and E, which were given different doses of Fe-HPH chelates, gradually increased as the dose provided increased; meantime, the SOD activity in Group E individuals was close to that of their counterparts belonging to Group B (positive control group). The following decreasing sequence on SOD activity in the liver of test groups was obtained: Group E > Group D > Group G > Group F > Group C.Concerning the muscle analysis, the SOD activities of rats in all groups (except for Group A) increased significantly or extremely significantly (*p* < 0.05 or *p* < 0.01) than those in Group A. The following decreasing effect on SOD activity in the muscle of test groups was observed: Group E > Group D > Group B > Group C > Group G > Group F. When compared with Group B individuals, the SOD activity in their counterparts belonging to Group E increased significantly (*p* < 0.05).

In summary, medium and high doses of Fe-HPH chelate showed a stronger effect on the increase of SOD activity in rats than HPH or Fe alone, and revealed similar or higher effects than GSH.

**Table 7:** Fe-HPH II chelate and superoxide dismutase (SOD) activity in rat blood, liver, and muscle.

Group	Animal Number	SOD Activity (U/gHb)		
		Whole Blood	Liver	Muscle
A	10	12976.36 ± 1904.76	70.38 ± 25.71	95.49 ± 23.73
B	10	14864.21 ± 1891.78 *	96.47 ± 11.32 *	233.74 ± 32.98 **
C	10	14002.16 ± 1212.85	60.74 ± 20.91 ^∆^	157.36 ± 17.39 *^,∆∆^
D	10	14789.09 ± 2464.06 *	76.29 ± 14.85	241.70 ± 39.54 **
E	10	15348.32 ± 1303.76 **	88.23 ± 19.23	299.46 ± 41.07 **^,∆^
F	10	13995.67 ± 865.21	69.14 ± 9.28	134.07 ± 20.02 *^,∆∆^
G	10	13366.07 ± 923.20	72.32 ± 7.11	156.37 ± 16.88 *^,∆^

Note: Relative to group A, * *p* < 0.05; ** *p* < 0.01; compared with group B, ^∆^ *p* < 0.05; ^∆∆^ *p* < 0.01.

### 3.6. Fe-HPH II Chelate and MDA Content in Rats

MDA is a lipid peroxidation metabolite found in the human body. It can generally serve as an indicator of the generation of free radicals and of damage to the membrane lipid bilayer [26]. When the MDA content of the body is excessive, there is a higher lipid peroxidation and free radical generation. Under such circumstances, it becomes easier to destroy the cell structure and functions, decrease the ATP production and cause fatigue.

As shown in Table 8, in whole blood, Groups B, D, and E had significantly or extremely significantly (*p* < 0.01 or *p* < 0.05) lower MDA content than Group A (negative control group). However, Groups C, F and G did not provide significant differences, being the mean reduction effect of Group F the weakest. In turn, the reduction effect on MDA content in whole blood of each group can be expressed by the following decreasing tendency: Group E > Group B > Group D > Group C > Group G > Group F. For the liver analysis, Groups D and E showed an obvious reduction effect on MDA content when compared with Group A (*p* < 0.05). The reduction effects of other four groups were not obvious, and the effect of Group F was the weakest. In turn, the reduction effect on MDA content in the liver of each group was according to the following decreasing sequence: Group E > Group D > Group B > Group C > Group G > Group F. In the muscle, except for Groups F, G and A, all groups reduced the MDA content significantly or extremely significantly (*p* < 0.01 or *p* < 0.05). The reduction effects in individuals corresponding to Groups D and E were higher than those of Group B (positive control group). There was a significant difference (*p* < 0.05) in the reduction effect on MDA content between Group B and Groups E and G. As a result, the reduction effect on the MDA content in the muscle can be expressed by the following decreasing sequence: Group E > Group D > Group B > Group C > Group F > Group G.

In summary, medium and high doses of Fe-HPH chelate revealed similar or higher effects on decreasing the MDA content than GSH. The reduction effect of HPH or Fe alone was always the weakest.

**Table 8:** Fe-HPH II chelate and malondialdehyde (MDA) content in rat blood, liver, and muscle.

Group	Animal Number	MDA Content (nmol/mL)		
		Whole Blood	Liver	Muscle
A	10	3.67 ± 0.69	13.13 ± 4.51	29.63 ± 3.52
B	10	1.96 ± 0.24 **	12.02 ± 0.11	18.09 ± 1.38 *
C	10	2.73 ± 0.87 ^∆^	12.54 ± 1.23	20.37 ± 2.16 *
D	10	2.06 ± 0.31 *	11.81 ± 0.19 *	17.54 ± 1.83 **
E	10	1.55 ± 0.17 **	10.67 ± 0.78 *	16.03 ± 1.82 **
F	10	3.02 ± 0.13 ^∆^	13.08 ± 0.13	25.33 ± 1.07 ^∆^
G	10	2.89 ± 0.66 ^∆^	12.88 ± 1.02	25.76 ± 1.12 ^∆^

Note: Relative to group A, * *p* < 0.05; ** *p* < 0.01; compared with group B, ^∆^ *p* < 0.05.

### 3.7. Fe-HPH II Chelate and GSH-Px Activity in Rat Livers

The enzyme GSH-Px scavenges free radicals, inhibits free radical reactions, prevents membrane lipid peroxidation, and preserves cell membrane structure and functions. Additionally, a profitable effect on fatigue prevention has also been pointed out [27]. As shown in Table 9, the GSH-Px activities of rats in Groups B, D and E were significantly or extremely significantly (*p* < 0.05 or *p* < 0.01) higher than those in Group A (negative control group). The increasing effect on GSH-Px activity of Group-E individuals was higher than that of their counterparts from Group B (positive control group). Groups C, F and G were visibly different (*p* < 0.05) from Group B, showing the following decreasing sequence: Group G > Group C > Group F.

In summary, the increasing effect on GSH-Px activity of Fe-HPH chelate (medium or high doses) was always stronger than that of HPH or Fe alone.

**Table 9:** Fe-HPH II chelate and the glutathione peroxidase (GSH-Px) activity and blood lactic acid.

Group	Animal Number	GSH-PxActivity	Blood lactic Acid Content (mmol/L)
A	10	41,138.05 ± 1193.34	8.01 ± 1.45
B	10	50,287.21 ± 2107.23 *	4.02 ± 0.33 **
C	10	43,739 ± 1880.26 ^∆^	7.23 ± 0.98 ^∆∆^
D	10	49,183.51 ± 1694.76 *	6.25 ± 0.71 *^∆^
E	10	53,177.32 ± 2350.17 **	4.01 ± 0.08 **
F	10	42,267.18 ± 933.15 ^∆^	7.10 ± 0.32 ^∆∆^
G	10	43,832.15 ± 1003.43 ^∆^	7.14 ± 0.80 ^∆∆^

Note: Relative to group A, * *p* < 0.05; ** *p* < 0.01; compared with group B, ^∆^ *p* < 0.05; ^∆∆^ *p* < 0.01.

### 3.8. Fe-HPH II Chelate and Blood Lactic Acid Content in Rats

The human body produces large amounts of lactic acid during strenuous exercise. When the lactic acid enters the bloodstream, the blood lactate concentration increases significantly, the hydrogen ion concentration increases, metabolic disorders become more pronounced, and the animal experiences fatigue [8]. As shown in Table 9, individuals from Groups B, D and E had significantly or extremely significantly (*p* < 0.01 or *p* < 0.05) lower lactic acid contents in the blood than their counterparts from Group A (negative control group). The decreasing effects of Groups C, F and G were not obvious, and the following decreasing sequence was observed: Group F > Group G > Group C. Groups C, D, F and G were significantly or extremely significantly (*p* < 0.05 or *p* < 0.01) different from Group B (positive control group). The decreasing effect on blood lactic acid of Group E was similar to that of Group B.

In summary, as the dose of Fe-HPH chelate included in the diet increased from low to high, the lactic acid levels in rats gradually became similar to those individuals given GSH. It is concluded that the effect of medium and high doses of Fe-HPH chelate was higher than that of HPH or Fe alone.

## 4. Conclusions

The chelation rate refers to the combination of ferrous ions with amino acids or peptides. The higher the chelation rates, the higher combinations of ferrous ions and amino acids or peptides were produced. Fe-HPH II chelate had the highest chelation rate in the three kinds of Fe-HPH chelates. In the amino acid composition analysis, the concentrations of amino acids related to anti-fatigue, anti-oxidation, and fatigue delay of Fe-HPH II chelate were all moderate or high. In the *in vitro* antioxidant activity assays, Fe-HPH II chelate revealed the strongest scavenging effects on hydroxyl radicals, DPPH and superoxide anion radicals when compared with the other two chelates; such a result was in agreement with the amino acid composition results. Accordingly, the Fe-HPH II chelate was chosen for the subsequent anti-fatigue animal experiment.

In animal experiments, Fe-HPH II chelate visibly increased haemoglobin regeneration efficiency (HRE) of rats, and significantly prolonged the exhaustive swimming time of rats. This kind of chelate also led to significant SOD activity increase in rats’ muscles and whole blood, showing an increasing trend in livers and visibly reduced the MDA content in rats’ livers, muscles, and whole blood, especially in the case of muscle and whole blood. Moreover, Fe-HPH II chelate showed a significant increasing effect on GSH-Px activity in rats’ liver and a significant reduction in blood lactic acid content. SOD and GSH-Px are recognized as primary antioxidant enzymes; their activities become weaker during chronic fatigue or other disease conditions, so that the improvement of their activities can help to avoid fatigue development. The anti-fatigue effect of HPH hydrolysate or Fe alone was far lower than that of medium and high dose of Fe-HPH chelate. It was concluded that the anti-fatigue ability of Fe-HPH chelate was due to the interactive effect between iron and HPH hydrolysate, and the ability was affected by the dose of Fe-HPH chelate, *i.e.*, the higher the dose provided, the stronger the anti-fatigue ability. Compared with the positive control substance (GSH), a high dose of Fe-HPH II chelate could lead to the similar effect or even better than GSH addition on the anti-fatigue development.

In conclusion, Fe-HPH II chelate revealed a profitable anti-fatigue effect in rats. This result may serve as a basis for isolation and identification of Fe-HPH II chelate, which showed the best anti-fatigue effects and also for the assessment of the ideal amino acid composition of purified peptide. The anti-fatigue mechanism also needs to be studied further.
